# Identification of 2-hydroxyisocaproic acid production in lactic acid bacteria and evaluation of microbial dynamics during kimchi ripening

**DOI:** 10.1038/s41598-017-10948-0

**Published:** 2017-09-07

**Authors:** Boyeon Park, Hyelyeon Hwang, Ji Yoon Chang, Sung Wook Hong, Se Hee Lee, Min Young Jung, Sung-Oh Sohn, Hae Woong Park, Jong-Hee Lee

**Affiliations:** World Institute of Kimchi, Gwangju, 61755 Republic of Korea

## Abstract

Lactic acid bacteria produce diverse functional metabolites in fermented foods. However, little is known regarding the metabolites and the fermentation process in kimchi. In this study, the culture broth from *Leuconostoc lactis*, a lactic acid bacterium isolated from kimchi, was analysed by liquid chromatography-tandem mass spectrometry and identified by the MS-DIAL program. The MassBank database was used to analyse the metabolites produced during fermentation. A mass spectrum corresponding to 2-hydroxyisocaproic acid (HICA) was validated based on a collision-induced dissociation (CID) fragmentation pattern with an identified *m/z* value of 131.07. HICA production by lactic acid bacteria was monitored and showed a positive correlation with hydroxyisocaproate dehydrogenases (HicDs), which play a key role in the production of HICA from leucine and ketoisocaproic acid. Interestingly, the HICA contents of kimchi varied with *Leuconostoc* and *Lactobacillus* content during the early stage of fermentation, and the addition of lactic acid bacteria enhanced the HICA content of kimchi. Our results suggest that HICA production in kimchi is dependent on the lactic acid bacterial composition.

## Introduction

Fermentation improves the long-term storage of food and increases the contents of active metabolites, thereby contributing to human health^1^. Various studies have shown that fermented foods exhibit enhanced health-promoting effects. Kimchi is a representative fermented vegetable-containing food product with health-promoting activity. Kimchi improves lipid profiles by lowering low-density lipoprotein levels and has been reported to exert antioxidant and anti-obesity effects^1–4^. Kimchi contains a variety of bacteria, among which *Lactobacillaceae* and *Leuconostocaceae* are the dominant lactic acid bacterial families. The antibacterial activities and probiotic features of lactic acid bacteria from kimchi have been extensively characterized^5, 6^. In addition, lactic acid bacteria isolated from kimchi exhibit antioxidant, immunomodulatory, antifungal, antibacterial, and anti-adipogenic activities^7–12^. Moreover, exopolysaccharides, vitamins, phenolic compounds, γ-amino butyric acid, mannitol, and organic acids from kimchi have been reported to originate from lactic acid bacteria^13–20^.

The unique process of kimchi fermentation depends mainly on the physiological and biochemical traits of metabolite processing by the bacteria, with bacterial population changes contributing to the flavour and taste of kimchi through the conversion of raw materials into organic acids, sugars, and diverse components. The identification of metabolites from kimchi is necessary to elucidate these fermentation mechanisms. The metabolites in kimchi from the bacterial associations have not been characterized. Research has focused on the molecules that originate from the raw materials used to produce kimchi, such as cabbage, garlic, pepper^21^. Therefore, it would be of great interest to characterize metabolite conversion by bacteria during kimchi fermentation.

To identify these molecules, a bacterial culture was subjected to ultra-performance liquid chromatography (UPLC) coupled with TripleTOF technology using IDA (information-dependent acquisition) mode, and the resultant mass spectrum was analysed with the MassBank mass-spectral database using MS-DIAL software^22^. A mono-isotopic parent ion peak at *m/z* 131.07 and fragment masses at *m/z* 85.06 and 69.03 had the highest MS2 similarities and were identified as 2-hydroxyisocaproic acid (HICA). HICA is reported to be a leucine metabolite of *Lactobacillus* sp. We therefore characterized and analysed HICA production by lactic acid bacteria isolated from kimchi and monitored HICA production in relation to the bacterial composition during kimchi fermentation.

## Results

### Identification of 2-hydroxyisocaproic acid from lactic acid bacteria

**Figure 1:**
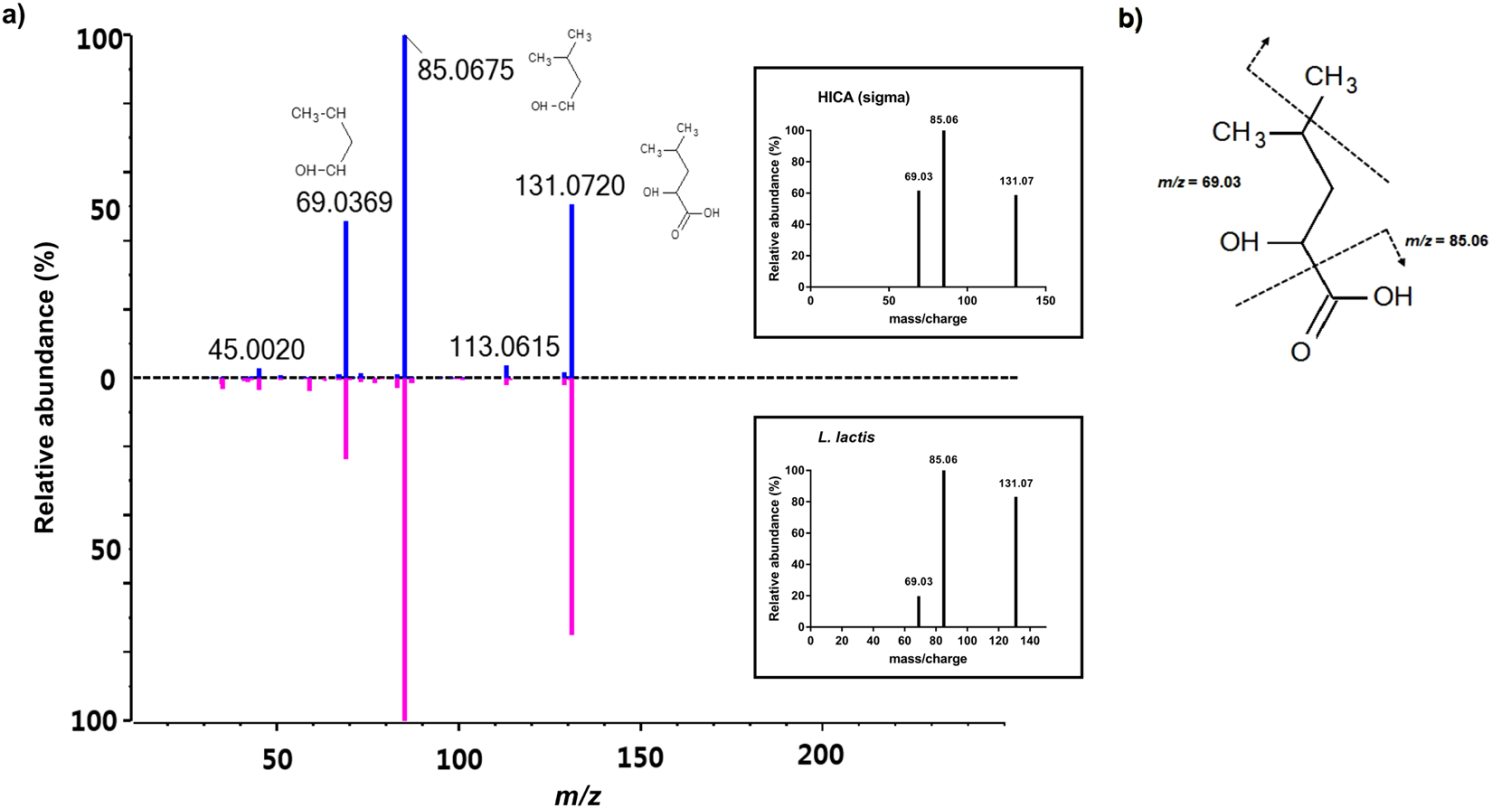
Identification of HICA from lactic acid bacteria using ultra-performance liquid chromatography combined with electrospray ionization time-of-flight MS. (**a**) Comparison of fragment-ion mass spectra of HICA with [M-H]^−^ at *m/z* 131.07 as the precursor ion. The fragment ion at *m/z* 131.07 at 1.4 min from HICA was obtained from a commercial supplier (the upper panel) or *L*. *lactis* (the lower panel). (**b**) Structural schematic of ionized HICA, which divided into three major fragments when the collision energy was −20 eV.

To investigate the metabolites of lactic acid bacteria isolated from kimchi, we first attempted to identify the metabolites of *Leuconostoc lactis* using a UPLC system coupled to mass spectrometry (MS/MS). The untargeted acquisition of metabolome data was processed by the MS-DIAL program with the MassBank database^22^. In the resulting spectrum, one molecule had an *m/z* value of 131.07 with fragment ions of *m/z* 85.06 and 69.03; these had the highest MS2 similarities, exactly matching the known MS/MS fragmentation patterns for HICA in MassBank (http://www.massbank.jp/en/ database.html) and METLIN (https://metlin.scripps.edu/metabo_advanced.php). To confirm the identity of the compound, collision-induced dissociation was performed to characterize the identified ion and compare the molecule with pure HICA (Sigma-Aldrich, St. Louis, MO, USA). The identified compound exhibited the same retention time and fragmentation pattern as pure HICA in product-ion MS mode (Fig. 1). The fragment ions at *m/z* 85.06 and 69.03 corresponded to neutral losses of 46.01 and 62.04 Da, respectively, from the precursor ion (*m/z* 131.07).

### Analysis of hydroxyisocaproate dehydrogenases (HicDs)

To investigate HicDs, which catalyse the conversion to HICA, the amino acid sequences of HicDs were obtained from the protein sequence collection in UniProt (http://www.uniprot.org/). *Leuconostoc lactis* and *Leuconoostoc mesenteroides* each have 1 annotated HicD protein. *Pediococcus pentosaceus* and *Lactobacillus sakei* do not have any proteins corresponding to HicDs, while *Lactobacillus brevis* has two and *Lactobacillus plantarum* has 2–4 HicD proteins (Table 1).

**Table 1:** HICA production and HicD proteins of lactic acid bacteria.

Strains	HICD detection^1^	Gene/Protein^2^
***Lactobacillus brevis***	⊚	
*L. brevis* ATCC 27350		*ldhD-3*/C2D5N4
		HMPREF0496_0286/C2CYB4
***Pediococcus pentosaceus***	○	na
***Lactobacillus plantarum***	⊚	
*L. plantarum* WCFS1		*hicD1*/F9UTU9
		*hicD2*/F9UN38
		*hicD3*/F9UQQ1
*L. plantarum* 16		Lp16_0961/R9X0G7
		Lp16_1847/R9X4I9
		Lp16_0312/R9WYL0
*L. plantarum* EDG-AQ4		N692_14205/T5JVU0
		N692_07995/T5JPG7
		N692_09230/T5JNS8
*L. plantarum* JCM 1149		*hiD2*/D7V8C8
		*hiD3*/D7VB10
*L. plantarum* DSM 16365		FD10_GL001795/A0A0R1UA88
		FD10_GL001842/A0A0R1UFN3
		FD10_GL000167/A0A0R1UV40
		FD10_GL002915/A0A0R1UXZ3
***Leuconostoc lactis***	⊚	
*L. lactis* ATCC 19256		AN225_00170/A0A0Q0YB06
***Lactobacillus sakei***	○	na
***Leuconostoc mesenteroides***	⊚	
*L. mesenteroides* J18		MI1_00275/A0A0N1S1U2
*L. mesenteroides* ATCC 19254		HMPREF0555_1660/C2KLZ4
*L. mesenteroides* P45		LH61_00190/A0A095BJX0
*L. mesenteroides* subsp. *dextranicum*		WZ78_04955/A0A0K9JDI8

^1^HICD was identified and measured using UPLC-ESI-TOF-MS/MS (○: > 20 μg/ml, ⊚: > 150 μg/ml).

^2^Gene/Protein IDs were obtained from the UniProt (http://www.uniprot.org/) protein database.

**Figure 2:**
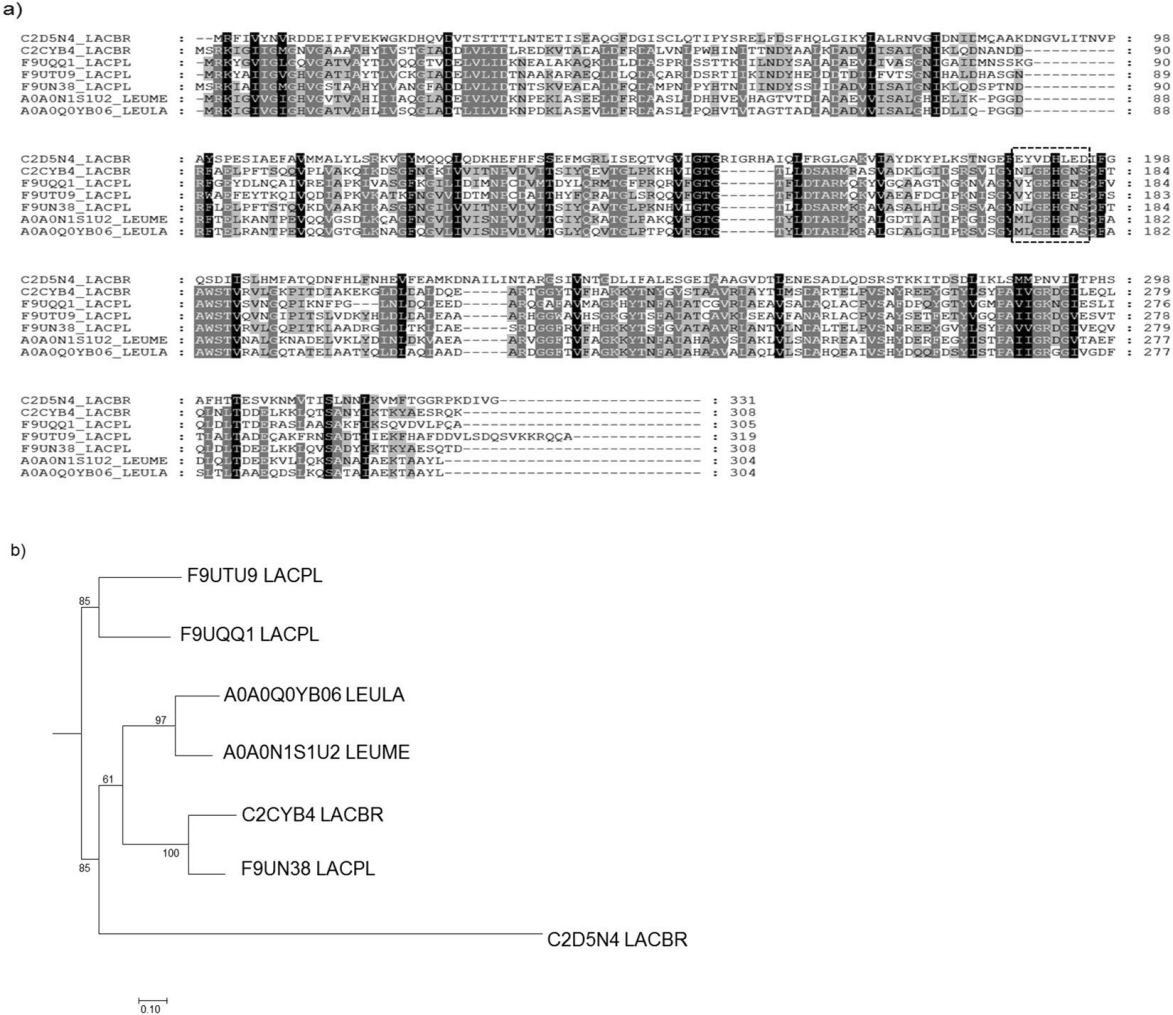
Comparison of hydroxyisocaproate dehydrogenases from lactic acid bacteria. **(a**) Alignments of hydroxyisocaproate dehydrogenases of lactic acid bacteria. The boxed region indicates the proton-donor active site. (**b**) Phylogenetic trees constructed from hydroxyisocaproate dehydrogenase protein sequences showing phylogenetic relationships. (LACPL: *Lactobacillus plantarum*; LEULA: *Leuconostoc lactis*: LEUME: *Leuconostoc mesenteroides*; LACBR: *Lactobacillus brevis*).

We compared the HicDs among the lactic acid bacteria and were able to classify them into *Lactobacillus* and *Leuconostoc* groups (Supplementary Fig. S1 and Fig. 2). For further investigation, we selected strains with reliable reference genomes (*L*. *plantarum* WCFS1^23^, *L*. *mesenteroides* J18^24^, *L*. *brevis* ATCC 27350, and *L*. *lactis* ATCC 19256) and aligned their HicD amino acid sequences using the ClustalW multi-alignment program to compare the core regions of the amino acid sequences (Fig. 2). The active domain was analysed by performing a ProSite scan-motif search (http://prosite.expasy.org/), which identified LGEHGNS as the proton-donor active site (Fig. 2a). The HicD amino acid sequences with the UniProt accession numbers A0A0N1S1U2 (*L*. *mesenteroides* J18) and A0A0Q0YB06 (*L*. *lactis* ATCC 19256) exhibited 75.33% similarity, whereas F9UN38 (*L*. *plantarum* WCFS1) and C2CYB4 (*L*. *brevis* ATCC 27350) exhibited 74.68% similarity. The other HicDs exhibited 40–55% similarities, except for C2D5N4 (*L*. *brevis* ATCC 27350), which showed 11% similarity.

### Quantification of HICA and *hicD* gene expression

**Figure 3:**
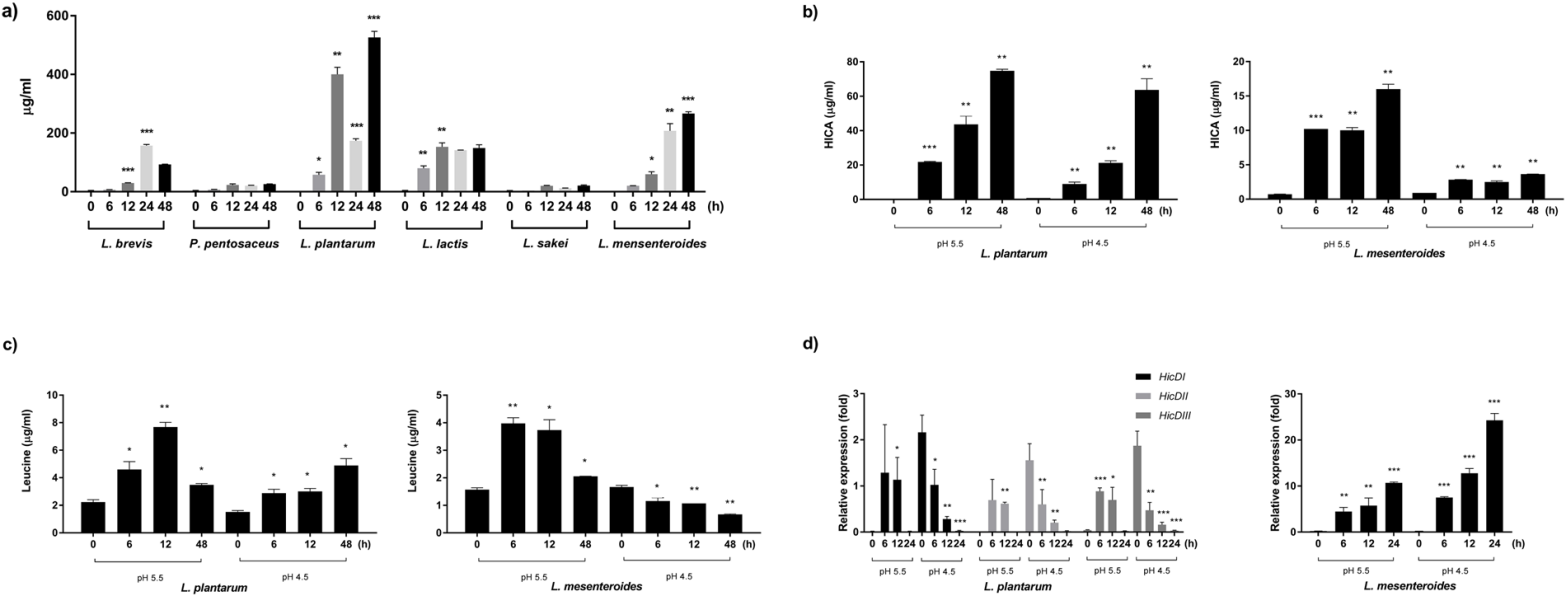
Confirmation of the HICA and leucine contents from lactic acid bacteria and the expression of *hicD* genes according to growth and MRS conditions. (**a**) Comparison of HICA production by six lactic acid bacteria, including *Lactobacillus brevis*, *P*. *pentosaceus*, *Lactobacillus plantarum*, *Leuconostoc lactis*, *Lactobacillus sakei*, and *Leuconostoc mesenteroides*. (**b**,**c**) Comparison of the change in HICA production and intracellular leucine content induced by *Lactobacillus plantarum* and *Leuconostoc mesenteroides* in MRS broths of pH 5.5 and 4.5 adjusted with lactic acid solution. (**d**) The transcription levels of the 2-hydroxyisocaproate dehydrogenase genes in *Lactobacillus plantarum* and *Leuconostoc mesenteroides* in MRS broths of pH 5.5 and 4.5 were determined via qRT-PCR. The mRNA expression values were normalized by the transcription levels in bacteria cultivated in MRS pH 6.2 according to growth time. Asterisks indicate significant differences (***P < 0.001; **P < 0.01; *P < 0.05).

For quantification of HICA content, a bacterial culture extract was analysed by UPLC combined with electrospray ionization time-of-flight MS (UPLC-ESI-TOF-MS/MS). Quantification of HICA was achieved by comparing the peak area of l-norvaline (internal standard) with the areas of relevant peaks from extracted-ion chromatograms (internal standard, *m/z* 116 > 116; HICA, *m/z* 131 > 85). The HICA content of *L*. *plantarum* reached 526 ± 20.9 μg/ml at 48 h (*P* = 0.00078) after cultivation, whereas *L*. *lactis* and *L*. *mesenteroides* produced 153.1 ± 13.7 (*P* = 0.004) and 266.9 ± 5.9 μg/ml (*P* = 0.0002) at 12 and 48 h, respectively (Fig. 3a). HICA content began to increase at 6 or 12 h with the growth of lactic acid bacteria. *L*. *plantarum* possesses three HicD proteins, and it produced approximately two fold more HICA than *L*. *lactis* or *L*. *mesenteroides* (Fig. 3a). However, although *L*. *brevis* possesses two HicD proteins, its HICA production was the lowest among the four lactic acid bacteria studied. This may be because one of its HicD proteins (C2D5N4) exhibited low similarity to the other HicDs (Fig. 2a and b). The HICA contents of *L*. *plantarum* and *L*. *mesenteroides* were positively correlated with the intracellular leucine content (Fig. 3b and c). In addition, the HICA content of *L*. *mesenteroides* increased despite growth suppression at pH 5.5 (Fig. 3b).

*hicD* gene expression from lactic acid bacteria was measured using quantitative real-time PCR. Bacteria were cultivated in MRS broth in which the original pH of 6.2 was adjusted to 4.5–5.5 with lactic acid. *hicD* gene expression increased as bacterial growth increased or as pH decreased (Fig. 3d and Supplementary Fig. S3). The three *hicD* homologues of *L*. *plantarum* all showed similar gene expression patterns. While *L*. *brevis* possesses two *hicD* genes, HMPREF0496_0286 (C2CYB4) did not exhibit an increase in gene expression with increasing bacterial growth (Supplementary Fig. S3).

**Figure 4:**
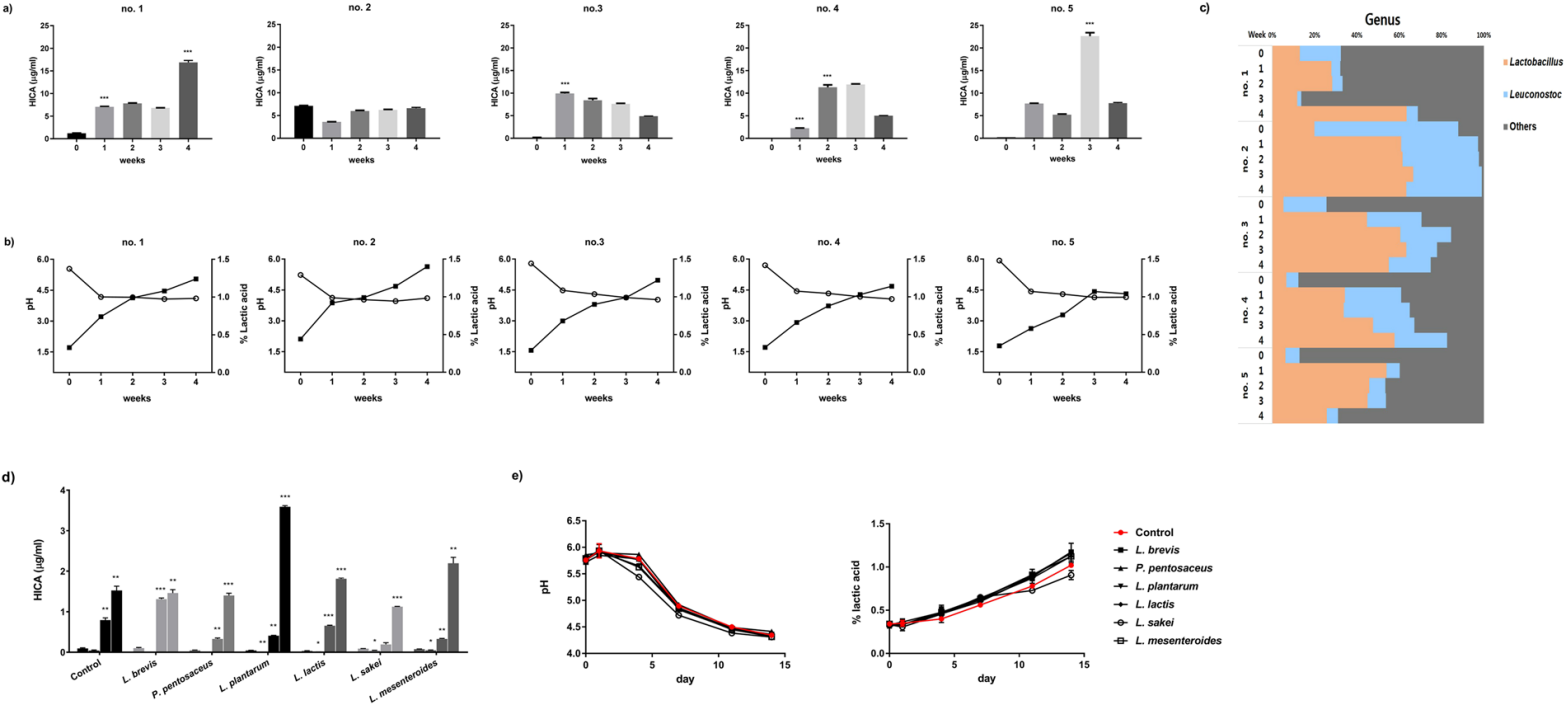
Properties of kimchi producing HICA obtained from a local market or treated with lactic acid bacteria starter. (**a**) Quantification of HICA production in commercial kimchi. The HICA content was determined after LC-MS/MS separation and analysis in multiple-reaction monitoring mode with measurements of the product ion. All kimchi samples were purchased from a local market. (**b**) Changes in pH and acidity in commercial kimchi. (**c**) Comparison of bacterial communities among commercial kimchi. *Lactobacillaceae* and *Leuconostocaceae* are dominant. (**d**) Quantification of HICA production in kimchi treated with lactic acid bacteria for 0, 4, 7, and 14 days. (**e**) Measurements of the pH and acidity of starter-inoculated kimchi. Asterisks indicate significant differences (***P < 0.001; **P < 0.01; *P < 0.05).

Next, we measured the HICA contents of kimchi obtained from a local market. The HICA contents of the kimchi samples began to increase after approximately 2 weeks and then decreased over a 3–4-week period. Kimchi HICA content ranged from 7.1 ± 0.1 to 22.6 ± 0.4 μg/ml during the period from 2 weeks to 4 weeks (Fig. 4a).

To investigate the association between HICA content and the kimchi microbiota, we examined the kimchi microbiota using metagenomic techniques and measured the acidity of each kimchi sample. As shown in Fig. 4b, acidity increased during kimchi fermentation, with all kimchi samples showing a similar pattern in which acidity increased from approximately 0.3% at the initial time point to approximately 1.2% after 4 weeks of fermentation. The overall HICA contents of kimchi increased (*P* < 0.05) after 1 week of fermentation, except for in one kimchi sample (no. 2), which nonetheless showed a similar acidity to those of the other kimchi samples. The microbiota of the kimchi was dominated by the *Lactobacillus* and *Leuconostoc* genera, in accordance with the presence of fermentation (Fig. 4c). Two kimchi samples (no. 1 and 2) showed relatively high abundances of lactic acid bacteria and higher HICA contents (1.2–7.1 μg/ml) than the other kimchi samples (0.1–0.2 μg/ml) at the initial time point (0 W).

To determine whether the addition of lactic acid bacteria contributes to the production of HICA in kimchi, we inoculated 10^7^ CFU/g bacteria into kimchi. Kimchi inoculated with *L*. *plantarum* and *L*. *mesenteroides* showed higher contents of HICA (2.2–3.5 μg/ml) (*P* < 0.01) than control kimchi samples (1.1–1.5 μg/ml) (Fig. 4d). The pH and acidity of inoculated kimchi were not dependent on HICA content (Fig. 4e).

## Discussion

In this study, we identified HICA from bacterial cultures of *L*. *lactis*, *L*. *plantarum*, *L*. *brevis*, and *L*. *mesenteroides* through an untargeted metabolomics approach. The amino acid derivative HICA (also known as leucic acid, 2-hydroxy-4-methylvaleric acid, and 2-hydroxy-4-methylpentanoic acid) is a protein-fermentation product of bacteria, such as lactobacilli. In addition, mammalian cells can metabolize HICA and use the metabolite for protein synthesis^25^. However, the generation of HICA in fermented vegetables and the lactic acid bacteria responsible for HICA production in fermented foods have not been investigated thoroughly.

A previous report showed that the *Lactobacillus amylovorus* DSM19280 (cereal isolate), *Lactobacillus brevis* R2Δ (porcine isolate), and *Lactobacillus reuteri* R29 strains produce HICA and characterized their antifungal activities^26^. HICA exhibits mild fungicidal or antibiotic activity against *Candida*, *Aspergillus*, *Staphylococcus*, and *Fusobacterium* ^27, 28^ and also shows activity against *Enterococcus faecalis* isolated from human teeth^29^. In a murine model, HICA attenuated inflammatory responses during *Candida* infection^25^. HICA can increase protein synthesis and improve muscle recovery after immobilization-induced atrophy^30^. The induction of protein synthesis is believed to occur through the activation of mammalian mTOR signalling, which is also activated during the innate inflammatory responses induced by bacteria, fungi, parasites, and viruses^30^.

HICA is formed by the transamination of leucine to 2-ketoisocaproic acid (KICA), followed by a reduction reaction of 2-KICA to 2-HICA, which is the end product of the leucine catabolism pathway. HicD is required for the latter reaction^27^. HICA is a typical constituent of human plasma, naturally circulating at a concentration of 0.25 ± 0.02 mmol/L^31^, and is found in muscle generally considered to have anti-catabolic activity^30^. HICA is also detectable in urine^32^ and other biological fluids^33–35^. HICA can inhibit various matrix metalloproteinase enzymes that are responsible for degrading connective and protein tissues^36^.

HICA production during bacterial growth was measured in multiple-reaction monitoring (MRM) mode during LC-MS/MS analysis. HICA production ranged from 153.1 to 526 μg/ml in *L*. *lactis*, *L*. *plantarum*, *L*. *brevis*, and *L*. *mesenteroides*. HICA production was lower in *L*. *sakei* and *P*. *pentosaceus* 21.4 and 26.1 μg/ml, respectively), which do not have HicDs.

These results agree well with both the numbers and similarities of HicDs in the bacterial strains. HicD was not identified in *P*. *pentosaceus* and *L*. *sakei*, and these two lactic acid bacteria strains showed reduced HICA production during growth. However, HicD belongs to the family of lactate dehydrogenases (Table S1), suggesting basal expression of HICA even in the absence of HicDs.

Previously, changes in the microbiota during kimchi fermentation were well characterized, and it was shown that *Lactobacillaceae* and *Leuconostocaceae* are the dominant microbial families in kimchi, with *Lactobacillus* species present during the later stages of fermentation and *Leuconostoc* species found during the early stages of kimchi fermentation^6, 37^. Even with varying degrees of acidity among the lactic acid bacteria, all cultures were acidic, and this increased in parallel with bacterial growth.

Therefore, we studied HICA production in kimchi and microbial populations to evaluate HICA production during kimchi fermentation. The HICA content in kimchi began to increase after 1 week, with the exception of that in kimchi sample no. 2 (Fig. 4b). Kimchi samples no. 1 and 2 had higher HICA contents at the starting time point but had low acidity and were composed predominantly of *Lactobacillus* and *Leuconostoc* (Fig. 4c). Nonetheless, HICA contents were consistent with changes in the microbiota despite the low acidity. Moreover, HICA contents in kimchi with added lactic acid bacteria support this finding. HICA contents were highest in the kimchi samples with *L*. *mesenteroides* and *L*. *plantarum* (Fig. 4d).

Acidity is used as a standard to evaluate the progress of fermentation in kimchi and other fermented foods, as acidity increases with the duration of kimchi fermentation. Leucine and ketoisocarproic acid are the main substrates in the HICA catabolic pathway in bacteria. Isovaleric acid is the final product of leucine oxidization, while reduction promotes the final product of isocaproic acid^38^. Acidic stress in *L*. *rhamnosus* and *L*. *reuteri* results in an increase in ABC-type dipeptide/oligopeptide transporter (dpp/opp) expression, which plays an important role in amino acid transport^39, 40^. A study into the *L*. *casei* amino acid uptake system suggested that leucine is transported by a proton drive motive force. Reductions in the proton motive force due to inhibitors or changes in pH decrease leucine transport^41^. In this study, we observed an increase in intracellular leucine, and this was positively correlated with HICA production (Fig. 3b,c). The fact that *hicD* expression was also increased at lower pH ranges supports the idea that the kimchi fermentation environment might be positively influenced by HICA production by lactic acid bacteria (Fig. 3d), especially by *Lactobacillus* and *Leuconostoc* during the initial kimchi fermentation process.

In conclusion, we found that the production of HICA as a leucine metabolite differed among lactic acid bacteria. These differences were mainly due to the core HicDs present in the lactic acid bacteria and to environmental stress during fermentation. These results also suggest the possible use of HICA as an indicator for fermented foods with lactic acid bacteria or related industrial processes.

## Materials and Methods

### Medium and bacterial culture conditions

All bacteria were isolated from homemade kimchi and identified by 16 S rDNA sequencing^42^. The identified 16 S rRNA gene sequences were deposited in NCBI GenBank under accession nos. KT759681 (*Leuconostoc mesenteroides* WiKim19), KX890131 (*Pediococcus pentosaceus* WiKim20), KX886794 (*Lactobacillus brevis* WiKim47), KX886799 (*Lactobacillus lactis* WiKim48), and KX886806 (*Lactobacillus sakei* WiKim49). *Lactobacillus plantarum* WiKim18 was reported previously^43^. All bacteria were cultivated in de Man, Rogosa, and Sharpe (MRS) broth.

### Preparation of samples and standard solutions

Bacterial culture samples were collected at 0, 6, 12, 24, and 48 hours after incubation at 30 °C and were centrifuged at 10,000 × *g* for 10 min; l-norvaline was added as an internal standard (final concentration: 100 μg/ml). The samples were extracted using a Sep-Pak C18 Light cartridge (Waters, Milford, MA, USA). Finally, the collected samples were lyophilized in a Speed-Vac and resuspended with distilled water for LC-MS/MS analysis. To prepare standard solutions, a stock solution of HICA was diluted serially to concentrations of 100, 300, 500, 800, and 1000 μM in 10 ml MRS broth. l-Norvaline was used as an internal standard at a concentration of 100 μg/ml in 10 ml MRS broth or bacterial culture supernatant. HICA and the internal standard were obtained from Sigma Aldrich (St. Louis, MO, USA) and used to generate standard curves for subsequent quantification. All solvents were LC-MS grade and were purchased from J. T. Baker (Phillipsburg, NJ, USA).

### UPLC-ESI-TOF-MS/MS conditions, HICA identification, and quantification

A TripleTOF 5600 plus instrument (SCIEX, Redwood City, CA, USA) coupled with an Acquity UPLC system (Waters) was used to characterize the metabolites and quantify the HICA contents of bacteria and kimchi. MS results were obtained at *m/z* 50–2000 in electrospray-negative mode with a spray voltage of −4.5 kV at a scan rate of 10 spectra/s. A reversed-phase column (Acquity UPLC BEH C18 column 2.1 × 100 mm, 1.7 μm particle size; Waters) was used to separate the compounds. The mobile phase consisted of distilled water (solvent A) and acetonitrile (solvent B) containing 10 mM ammonium acetate at a flow rate of 0.5 ml/min. The UPLC gradient program was as follows: (1) 95% solvent A from 0 to 0.5 min, (2) a linear gradient from 70% to 45% over 18.4 min, (3) 10% solvent A for 5 min. The total run time was 30 min per sample, and the injection volume was 2 μl. All experiments were performed in triplicate, and the data were processed using SCIEX PeakView 1.2 and MultiQuant 2.1 software (ABsciex). Peak picking and alignment were performed using MS-DIAL (ver. 1.98)^22^. Representative MS/MS spectra were exported in abf format for MS-DIAL, and compound identification was performed against MS/MS libraries including MassBank (MassBank_MSMS_Neg_Rev173_vs1)^44^ and ReSpect (Respect_20120925_ESI_Negative_MSMS)^45^. HICA from lactic acid bacteria and kimchi was quantified in MRM mode, using the following transitions: HICA, *m/z* 131.0 > 85.0; l-norvaline, *m/z* 116.

### HicD sequence analysis

The amino acid sequences of HicD proteins from lactic acid bacteria were obtained from UniProt^46^ for *L*. *brevis* ATCC 27350 (C2CYB4, C2D5N4), *P*. *pentosaceus* (na), *L*. *plantarum* WCFS1 (F9UTU9, F9UN38, F9UQQ1), *L*. *lactis* ATCC 1956 (A0A0Q0YB06), *L*. *sakei* (na), and *L*. *mesenteroides* J18 (A0A0N1S1U2). The amino acid sequences were aligned and compared using the ClustalW program. Amino acid sequence similarities were analysed, and phylogenetic trees were generated using the CLC Genomics Workbench v7.5 program (Qiagen, Redwood City, CA, USA).

### Quantitative real-time PCR of *hicD* expression

*hicD* gene expression levels from lactic acid bacteria were measured using quantitative real-time PCR. The bacteria were cultivated in MRS broth. The pH of the MRS broth (pH 6.2) was adjusted to pH 4.5–5.5 with lactic acid. The bacterial cells were harvested and lysed for total RNA extraction using TRIzol (Invitrogen, Carlsbad, CA, USA) according to the manufacturer’s instructions. cDNA was generated and RT-PCR was performed using TOPreal™ qPCR 2X PreMix (Enzynomics, Daejeon, Republic of Korea). Relative expression levels were calculated and normalized to that of the 16 S rRNA gene. Primers were designed based on nucleotide sequences from *L*. *lactis* A0A0Q0YB06 (*AN225_0017*); *L*. *brevis* C2D5N4 and C2CYB4 (*ldhD3* and *HMPREF0496_0286*, respectively); *L*. *mesenteroides* A0A0N1S1U2 (*MI1_00275*); and *L*. *plantarum* F9UTU9, F9UN38, and F9UQQ1 (*hicd1*, *hicd2*, and *hicd3*, respectively) (Table S2).

### Comparison and analysis of metagenomic data

Metagenomic DNA was isolated from commercial kimchi and analysed by sequencing at Chunlab, Inc. (Seoul, Korea) using an Illumina MiSeq sequencing system (Illumina, San Diego, CA, USA) in accordance with the manufacturer’s instructions.

The taxonomic classification for each read was determined using the EzTaxon-e database (http://eztaxon-e.ezbiocloud.net). The richness and diversity of samples were confirmed by Chao1 estimation and the Shannon diversity index at a 3% distance. To compare operational taxonomic units (OTUs) among samples, shared OTUs were identified by XOR analysis using the CL community program (Chunlab Inc., Seoul, Korea).

## Electronic supplementary material

Supplementary information
