# Supplementary material for: Identification of 2-hydroxyisocaproic acid production in lactic acid bacteria and evaluation of microbial dynamics during kimchi ripening
Source: Sci Rep. 2017 Sep 7;7:10904. doi: 10.1038/s41598-017-10948-0 (PMC5589888; doi:10.1038/s41598-017-10948-0)
Supplement: Supplementary file 1 — Supplementary information [file 41598_2017_10948_MOESM1_ESM.pdf]

Supplementary information

Identification of 2-hydroxyisocaproic acid production in lactic acid bacteria and evaluation of microbial dynamics during kimchi ripening

Boyeon Park†, Hyelyeon Hwang†, Ji Yoon Chang, Sung Wook Hong, Se Hee Lee, Min Young Jung, Sung-Oh Sohn, Hae Woong Park, and Jong-Hee Lee*

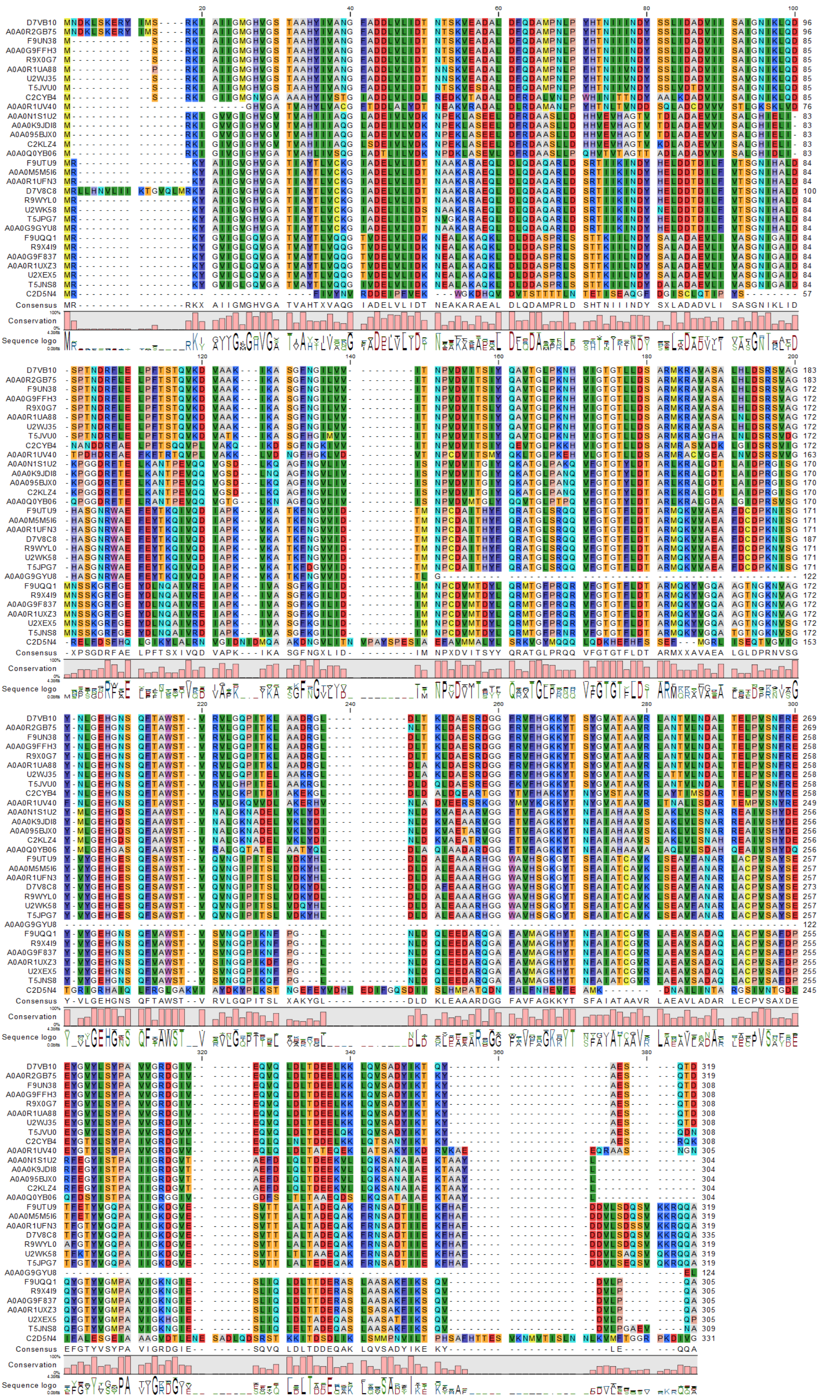

Supplementary figure S1. The multiple sequence alignment of 2-hydroxyisocaproate dehydrogenase of lactic acid bacteria using the ClustalW multi-alignment program

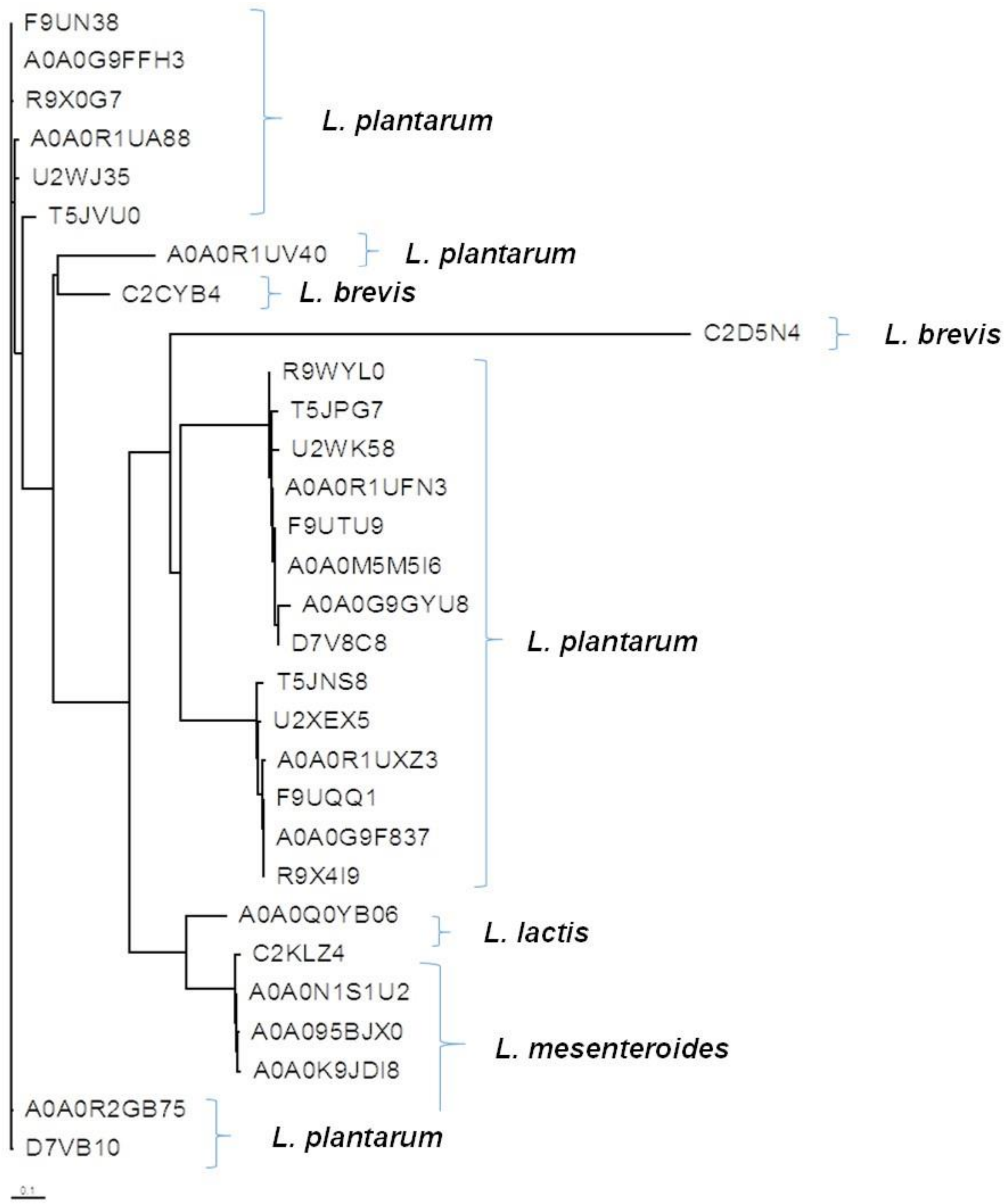

Supplementary figure S2. The phylogenetic trees constructed from 2-hydroxyisocaproate dehydrogenase amino acid sequence obtained Uniprot (www.uniprot.org/). The bar indicates 10% sequence divergence.

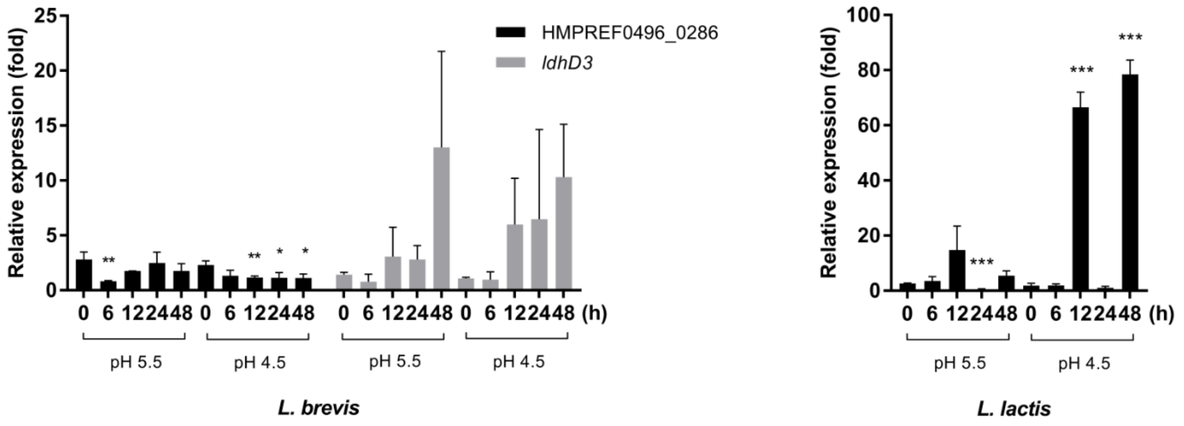

Supplementary figure S3. The transcription levels of the 2-hydroxyisocaproate dehydrogenase genes in *L. brevis* and *L. lactis* in MRS of pH 5.5 and 4.5 condition were determined via qRT-PCR. The mRNA expression values were normalized by the transcription levels by the bacteria cultivated in MRS (pH 6.2). Asterisks indicate significant differences (**P* < 0.001; ***P* < 0.01; **P* < 0.05).**

Supplementary table S1. Conserved domain structure of HicD identified in NCBI search

Amino acid sequence of HicD of *Leuconostoc lactis* was compared BLASTP database

Name	Accession	Description	Interval	E-value
HicDH_like	cd05291	L-2-hydroxyisocapronate dehydrogenases and some bacterial L-lactate dehydrogenases	3-304	6.39 E-149
Ldh_1_N	pfam00056	lactate/malate dehydrogenase, NAD binding domain	4-145	4.04 E-30
L-LDH-NAD	TIGR01771	L-lactate dehydrogenase	7-299	5.50 E-96
Mdh	COG0039	Malate/lactate dehydrogenase	3-299	1.32 E-85
ldh	PRK00066	L-lactate dehydrogenase	1-300	3.45 E-73

Supplementary Table S2. The primers for the real-time RT-PCR

**Species**	**Primers**	**Sequence**	**Reference**
Lactobacillus plantarum	LPWCFS_hicD1	5'-ATTCACGCGTTAGACCATGC-3'<br>5'-CTGTTGGCGTGATAATCCCG-3'	In this study
	LPWCFS_hicD2	5'-GCGACGTCTTTGACTTGTGT-3'<br>5'-CTTTGCCGATGACCTTGTCC-3'	
	LPWCFS_hicD3	5'-ATCGGACACCACAAGTAGCA-3'<br>5'-GTATGTCGGACAAGCAGCTG-3'	
Lactobacillus brevis	HMPREF0496_0286	5'-GATTGGACTTGGATGCGCTT-3'<br>5'-TGCTCTAAAATGCCGTCACG-3'	In this study
	Brevis ldhD3	5'-TTGGCGTTGAGAAATGTCGG-3'<br>5'-TTCACTGATTAGACGCCCCA-3'	
Leuconostoc lactis	Lactis_AN225_00170	5'-AAGACACCTTGAAAACCCGC-3'<br>5'-TTGATTTTCGTGATGCCGCT-3'	In this study
Leuconostoc mesenteroides	LM _LH61_00190	5'-GCGTGTACTTCGACATGATG-3'<br>5'-TTGTTGGCATTGGACATGTTG-3'	In this study
Lactobacillus plantarum<br>*Lactobacillus brevis*<br>*Leuconostoc lactis*	16S rRNA	5'-AGCAGTAGGGAATCTTCCA-3'<br>5'-CAC CGC TAC ACA TGG AG-3'	Walter *et al.* (2001)<br>Heilig *et al.* (2002)
Leuconostoc mesenteroides	16S rRNA	5'-AGC GTT ATC CGG ATT TAT TG-3'<br>5'-CTA CGC ATT CCA CCG CTA CA-3'	Minghui Yan *et al.* (2016)
